# Plasma miR-10a: A Potential Biomarker for Coronary Artery Disease

**DOI:** 10.1155/2016/3841927

**Published:** 2016-05-22

**Authors:** Liyun Luo, Bairong Chen, Songbiao Li, Xiaoliang Wei, Tianmin Liu, Yin Huang, Xiufang Lin

**Affiliations:** Department of Cardiology, The Fifth Affiliated Hospital of Sun Yat-sen University, Zhuhai, Guangdong 519000, China

## Abstract

*Aims*. MicroRNAs (miRNAs) are involved in the pathogenesis of coronary artery disease (CAD). The objective of this study is to determine plasma levels of miR-10a in CAD and analyze its association with the severity of CAD.* Materials and Methods*. Plasma miR-10a levels in 60 CAD patients including stable angina pectoris (SAP) (*n* = 29), unstable angina pectoris (UAP) or non-ST elevation myocardial infarction (MI) (NSTEMI) (*n* = 17), or ST elevation MI (STEMI) (*n* = 14) and 20 non-CAD subjects were assessed by real-time polymerase chain reaction (qRT-PCR), and associations of miR-10a levels with risk factors of CAD and its severity were analyzed.* Results*. The qRT-PCR results showed that plasma miR-10a levels were decreased in CAD patients, and CAD with high SYNTAX scores or STEMI was significantly associated with lower miR-10a levels.* Conclusions*. Lower plasma miR-10a levels were negatively associated with the presence as well as severity of CAD, and plasma miR-10a can act as a potential biomarker for estimating the presence and severity of CAD.

## 1. Introduction

Coronary artery disease (CAD) is currently one major cause of death in the world [1, 2]. CAD is mainly caused by atherosclerosis, which is considered as a chronic inflammation in response to cholesterol accumulation in the arterial wall [3]. Therefore, biomarkers that can predict the presence for early atherosclerotic process and CAD are desirable. MicroRNAs (miRNAs) are ~22 nucleotides long noncoding RNAs, known to inversely regulate their target gene expression at the posttranscriptional level by interacting with the 3′-untranslated region (3′-UTR) [4]. Accumulating evidence shows that miRNAs play important roles in the pathogenesis of atherosclerosis and CAD [4], and circulating miRNAs could be useful as novel biomarkers for the diagnosis of these diseases [5, 6].

Previous study has suggested that endothelial miR-10a was lower in the atherosusceptible regions of the inner aortic arch and aortorenal branches than elsewhere, and miR-10a can regulate proinflammatory phenotypes in atherosclerosis susceptible endothelium both in vivo and in vitro [7]. Although the role of miR-10a in the formation of atherosclerotic plaque has been reported, expression of different circulating miR-10a in CAD patients has not been studied yet.

In the present study, we aimed to determine plasma miR-10a levels in CAD patients and investigate the association between plasma miRNA-10a level and the severity of CAD.

## 2. Methods

### 2.1. Study Subjects

A total of 80 consecutive patients who underwent diagnostic coronary angiography for chest pain evaluation during January 2015 to July 2015 at Department of Cardiology, the Fifth Affiliated Hospital of Sun Yat-sen University, were enrolled into this study. In the whole populations studied, 60 subjects have been diagnosed with CAD, and subjects with no angiographic evidence of CAD were designed as non-CAD control (*n* = 20). Patients with CAD were furtherly grouped into either stable angina pectoris (SAP) (*n* = 29), unstable angina pectoris (UAP), or non-ST elevation myocardial infarction (MI) (UAP/NSTEMI) (*n* = 17) and ST elevation MI (STEMI) (*n* = 14) according to the ACC/AHA classification of the coronary tree. All subjects including patients and controls with a history and clinical features of acute or chronic infectious disease, lung diseases, liver disease and kidney disease, malignant tumor, and autoimmune diseases and patients who took anti-inflammatory drugs were excluded from this study. This study was approved by the human ethics committee of Sun Yat-sen University. All volunteers provided written informed consent, and the procedure was conducted in adherence with all applicable state and university guidelines.

### 2.2. RNA Isolation

Venous blood samples were obtained via antecubital venipuncture. Whole blood (5 mL) was collected in EDTA-containing tubes and then centrifuged (12,000 ×g for 10 min at 4°C). Supernatant was collected and centrifuged (12,000 ×g for 10 min at 4°C). Plasma was obtained and was rapidly subjected to RNA extraction. miRNA in plasma was isolated by the use of miRNeasy Plasma Kit (Qiagen, Hilden, Germany) in accordance with the manufacturer's protocol.

### 2.3. Quantitative Reverse-Transcription PCR

The expression levels of miR-10a in 60 patients with CAD and 20 control subjects were quantified by using a miScript SYBR Green PCR Kit (Qiagen, Germany). Monitoring of miRNA-derived PCR products was performed on a Light Cycler (Bio-Rad) and normalized to RNU6B. The 2^−ΔΔCt^ method was used for analysis of miR-10a expression (defined as fold change). The relative expression of miR-10a was calculated by the 2^−ΔΔCt^ (ΔCt = Ct^miR-10a^ − Ct^RNU6B^; ΔΔCt = ΔCt^sample^ − ΔCt^control^). All samples were repeated for trice.

### 2.4. Statistics

All statistical analyses were performed by using SPSS 16.0 (SPSS Inc., Chicago, IL). All continuous variables are expressed as the means ± SD. ANOVA or Student's *t*-tests were used for statistical analyses. *χ* ^2^ test was used to compare categorical variables. Pearson correlation coefficient was calculated for continuous variables. A *P* value < 0.05 was considered significant.

## 3. Results

### 3.1. Basic Clinical Characteristics of CAD Patients and Non-CAD Controls

Basic clinical characteristics of the whole subjects studied in this study were shown in Table 1. Among 80 subjects, 60 patients were found to have angiographically significant CAD and 20 patients were found not to have angiographically significant CAD, who grouped into non-CAD control. The CAD patients included SAP (*n* = 29), UAP/NSTEMI (*n* = 17), and STEMI (*n* = 14). *χ* ^2^ tests were used to compare categorical variables, and Student's *t*-tests were used to compare numerical variables. There were no substantial differences in age, gender, family history of CAD, and complication with diabetes between CAD group and control group (non-CAD patients). However, CAD group had significant higher BMI as well as obvious higher percentage of smokers, hypertension, and dyslipidemia.

### 3.2. Plasma miR-10a Levels Were Downregulated in CAD

**Figure 1:**
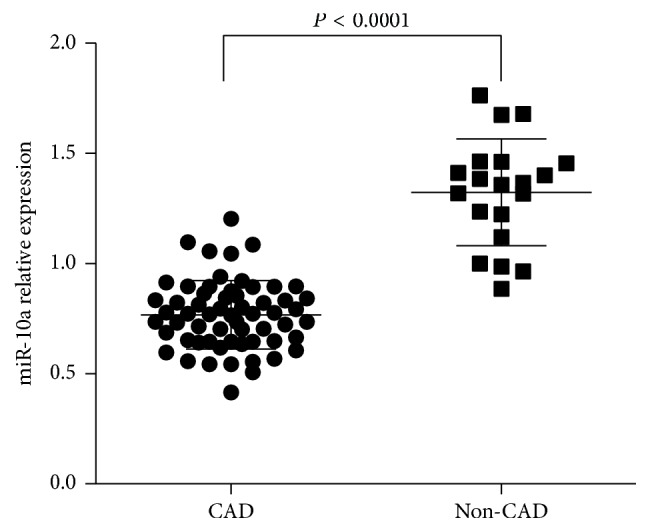
Plasma miR-10a levels in CAD patients and non-CAD controls. Plasma miR-10a levels were detected in CAD patients compared with non-CAD controls by qRT-PCR. The expression levels of miR-10a were normalized to RNU6B. *P* values were calculated using Student's *t*-test.

We determined the levels of miR-10a in the plasma of CAD patients and non-CAD subjects (controls). We designated the expression level of miR-10a in one non-CAD subject as 1, and other samples were compared with it. Student's *t*-tests were used for statistical analyses. The results showed that plasma miR-10a levels were decreased in CAD patients as compared with non-CAD controls (Figure 1).

### 3.3. Plasma miR-10a Levels Were Associated with Risk Factors in CAD Patients

We then analyzed the relationships between plasma miR-10a levels and risk factors in CAD patients. We set the two cut-off values of miR-10a expression levels in CAD patients as means − SD of miR-10a expression in non-CAD subjects. The relative expression of miR-10a < 0.838 was considered lower-expression. And *χ* ^2^ test was used to compare categorical variables. When comparing numerical variables, we defined them as categorical variables and then analyzed them by using *χ* ^2^ test. The cut-off values of numerical variables were shown in Table 2. The statistical analysis showed that there was no significant association of clinical variables and miR-10a (Table 2).

### 3.4. Plasma miR-10a Levels Were Associated with the Severity of CAD

**Figure 2:**
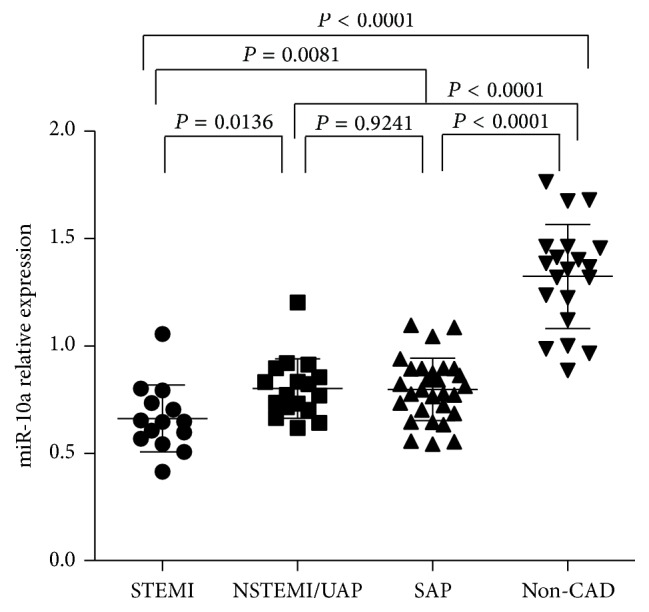
Plasma miR-10a levels among different clinical presentation of CAD. Plasma miR-10a levels were detected among different clinical presentation of CAD, including STEMI (*n* = 14), UAP/NSTEMI (*n* = 17), or SAP (*n* = 29), by qRT-PCR. The expression levels of miR-10a were normalized to RNU6B. *P* values were calculated using Student's *t*-test.

**Figure 3:**
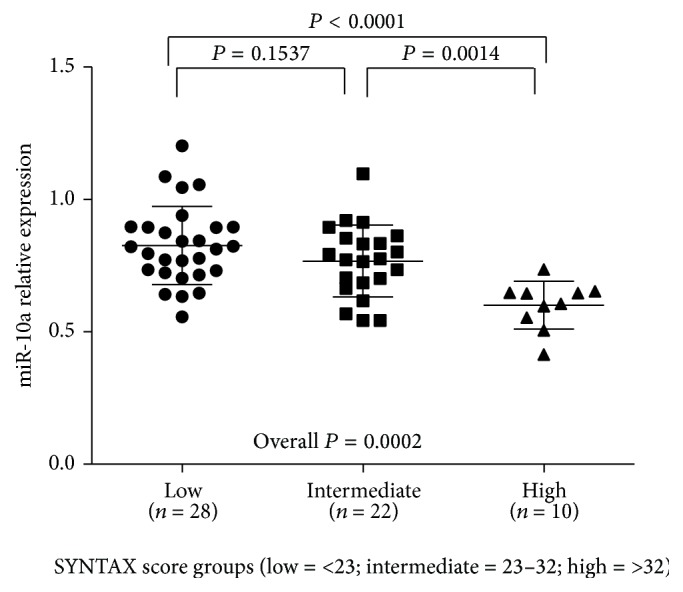
Plasma miR-10a levels among different SYNTAX groups. Plasma miR-10a levels were detected among different SYNTAX groups, including low (SYNTAX score < 23; *n* = 28), intermediate (SYNTAX score 23–32; *n* = 22), and high (SYNTAX score > 32; *n* = 10), by qRT-PCR. The expression levels of miR-10a were normalized to RNU6B. *P* values were calculated using Student's *t*-test. Overall *P* values were calculated using one-way ANOVA.

**Figure 4:**
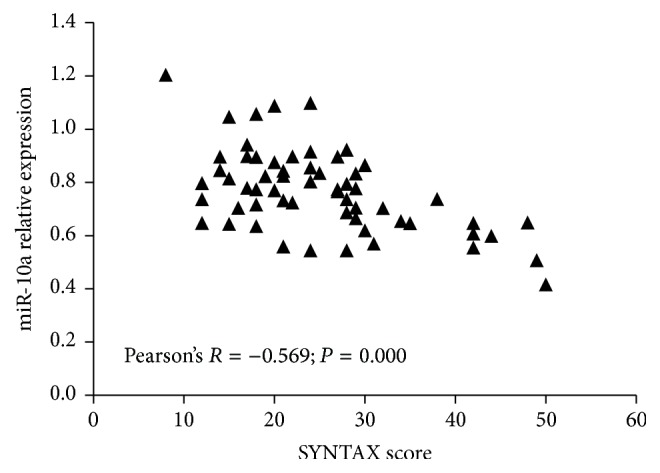
Correlation between SYNTAX score and plasma miR-10a. miR-10a was negatively associated with SYNTAX score in CAD patients.

To further evaluate the association between plasma miR-10a levels and the severity of CAD, we performed a subanalysis in CAD population (*n* = 60). Severity of CAD was evaluated with the type of CAD and SYNTAX score in this study. Firstly, we analyzed plasma miR-10a level in different groups including STEMI, UAP/NSTEMI, or SAP.  Figure 2 showed that, in STEMI group, miR-10a expression levels were downregulated as compared with UAP/NSTEMI and SAP group, but no significant difference was detected between UAP/NSTEMI and SAP group. On the basis of their SYNTAX scores, CAD patients were again divided into three groups: low (SYNTAX score < 23; *n* = 28), intermediate (SYNTAX score 23–32; *n* = 22), and high (SYNTAX score > 32; *n* = 10) and the plasma miR-10a levels in each group were analyzed. The results showed that patients in group with high SYNTAX score had significantly lower miR-10a levels, and no significant difference was observed between groups with low and intermediate SYNTAX score (Figure 3). Lower plasma miR-10a levels were negatively associated with higher SYNTAX scores (Pearson's *R* = −0.569; *P* = 0.000) (Figure 4). These results indicated decreased plasma miR-10a levels with increase in severity and complexity of CAD.

## 4. Discussion

MicroRNAs (miRNAs) are a class of ~22 nucleotides long noncoding RNAs, which regulate gene expression at the posttranscriptional level by binding with the 3′-untranslated region (3′-UTR) of their target genes [4]. miRNAs have been demonstrated to play vital roles in initiation and development of many diseases, such as cancers, autoimmune diseases, and CAD [4, 8, 9]. Recent studies have also shown that circulating miRNAs can act as novel biomarkers for the diagnosis and prognosis of these diseases [5, 6, 8]. CAD is currently considered as one major cause of death worldwide [1, 2]. CAD is mainly driven by atherosclerosis, which is characterized as a chronic inflammation [3]. Therefore, it is important to identify potential biomarkers of estimating the presence and the severity of CAD.

miR-10a had been found to be lower in the atherosusceptible regions of the inner aortic arch and aortorenal branches than elsewhere and to regulate inflammatory responses in atherosclerosis susceptible endothelium [7]. CAD is mainly caused by atherosclerosis, which is considered as a chronic inflammation in response to cholesterol accumulation in the arterial wall. Therefore, we speculated that aberrant miR-10a expression may be involved in CAD. Several studies have shown that circulating miRNAs can serve as potential biomarkers for CAD [5, 6]. In the present study, we demonstrated that plasma miR-10a levels were significantly downregulated in CAD patients, as compared with non-CAD controls. Our findings also indicated that downregulated miR-10a levels are correlated with the severity of CAD. Furthermore, we found that lower miR-10a levels were negatively associated with the severity of CAD, especially patients with STEMI and high SYNTAX score, which indicates that miR-10a may serve as potential biomarkers of the severity of CAD.

Exosomes are small membrane-bound vesicles secreted by most cell types, including endothelial cells, blood cells, immune cells, and tumor cells [10]. Exosomes contain various functional proteins, mRNAs, and miRNAs that could be used for diagnostic and therapeutic purposes in many diseases, such as CAD [10]. Herein, we demonstrated that plasma miR-10a is downregulated in CAD. Presumably the plasma miR-10a is in the plasma exosomes. Because the coronary tissue accounts for only a tiny fraction of the systemic vascular contribution of plasma exosomes, it is likely that the data of our study may not reflect the association of miR-10a and CAD. However, all subjects including patients and controls with a history and clinical features of acute or chronic infectious disease, lung diseases, liver disease and kidney disease, malignant tumor, and autoimmune diseases and patients who took anti-inflammatory drugs were excluded from this study. In addition, our team also determined the expression levels of plasma miR-10a in patients with heart failure and peripheral arterial disease, but no significant difference was observed between them and healthy controls (the results were not shown in the text). So we estimated plasma miR-10a as a biomarker of CAD. Certainly, further studies that indicate the association of miR-10a and CAD directly are required.

Our study had several limitations. Firstly, it was a single-center study which involved a small sample size, and the distribution of patients in CAD and non-CAD groups was uneven. Therefore, the results of this study might be interpreted with caution, and large-scale multicenter studies would be required to further illustrate miR-10a as a potential marker in CAD. Secondly, in this study we could not clarify the mechanisms of association between lower miR-10a levels in the plasma and CAD severity, and so some biological studies are also required.

In conclusion, plasma miR-10a levels are significantly lower in patients with CAD, when compared with non-CAD subjects, and this decrease is negatively associated with the severity of CAD. Our study may provide further evidence for clinical implications in the diagnosis of CAD.

## Figures and Tables

**Table 1:** Clinical characteristics in the whole population.

	CAD patients (*N* = 60)	Non-CAD control (*N* = 20)
Ages (years)	64.7 ± 8.7	63.4 ± 9.9
Gender (male), *n* (%)	45 (75)	14 (70)
Body mass index, kg/m^2^	25.4 ± 3.1^*∗*^	21.9 ± 3.4
Smoking, *n* (%)	42 (70)^*∗*^	7 (35)
Family history of CAD, *n* (%)	14 (23.3)	4 (20)
Hypertension, *n* (%)	52 (86.7)^*∗*^	5 (25)
Diabetes, *n* (%)	16 (26.7)	4 (20)
Dyslipidemia, *n* (%)	42 (70)^*∗*^	4 (20)

^*∗*^ *P* < 0.05.

**Table 2:** Correlation of miR-10a expression with clinical characteristics in CAD patients.

Variable	Normal expression	Low expression	*χ* ^2^	*P*
Total (*N* = 60)				
Sex			0.423	0.515
Male (45)	15	30		
Female (15)	3	12		
Age (years)			1.188	0.276
≤60 (19)	8	11		
>60 (41)	10	31		
Body mass index			0.060	0.8061
≥24 (42)	13	29		
<24 (18)	5	13		
Smoking			3.178	0.075
Yes (42)	16	26		
No (18)	2	16		
History of family			0.000	1.000
Yes (14)	4	10		
No (46)	14	32		
Diabetes mellitus			0.037	0.848
Yes (16)	4	12		
No (44)	14	30		
Hypertension			0.007	0.934
Yes (52)	15	37		
No (8)	3	5		
Dyslipidemia			3.178	0.075
Yes (42)	16	26		
No (18)	2	16		
